# 1440. Meta-regression Analysis of Worldwide Herpes Zoster Incidence

**DOI:** 10.1093/ofid/ofab466.1632

**Published:** 2021-12-04

**Authors:** Barbara P Yawn, Andrea Callegaro, Kyle Fahrbach, Binod Neupane, Hilde Vroling, Desiree Van Oorschot, Desmond Curran

**Affiliations:** 1 University of Minnesota, Minneapolis, Minnesota; 2 GSK, Rixensart/Wavre, Belgium, Rixensart/Wavre, Brabant Wallon, Belgium; 3 Evidera, Waltham, Massachusetts; 4 Pallas Health Research and Consultancy, Rotterdam, Zuid-Holland, Netherlands; 5 GSK Vaccines, Wavre, Vlaams-Brabant, Belgium; 6 GSK, Wavre, Vlaams-Brabant, Belgium

## Abstract

**Background:**

Many studies have been conducted worldwide to estimate Herpes Zoster (HZ) incidence rates and temporal trends. We systematically reviewed and synthesized studies of HZ incidence rates in the general population using meta-analysis models.

**Methods:**

A random-effects meta-analysis was conducted to estimate HZ incidence from a published worldwide systematic literature review (SLR) including only individuals aged 50 years and older. Meta-regression was used to explore if variability in incidence rates could be explained by a combination of study-specific characteristics in the base model: age, gender, continent and year of data collection. The impact of adding additional covariates: case detection, case definition, study design, incidence type, patient type and latitude to the base model was also assessed.

**Results:**

65 out of 69 studies from the SLR, were included in the analysis: 27 from Europe, 20 from North America, 11 from Asia and 7 from Oceania. There was much variability in study methodology and outcomes. Heterogeneity of incidence rates was greatest across studies conducted in Asia. Meta-analysis results showed that: incidence increased with age; was lower in males compared to females; was lower in Europe and North America compared to Asia and Oceania; and increased from the period prior to 2003 to the period after 2003. The final meta-regression model included continent, year of data collection, gender, age, cubic and quadratic terms for age, as well as an age x gender interaction term. The age x gender interaction suggests that the difference in incidence between males and females is greater in younger ages (e.g. 50-59), whereas in older age groups (e.g. 80+) incidence rates are similar between males and females. None of the additional covariates contributed significantly to the model. It was estimated that 15.5 million HZ cases occurred in 2020 worldwide in individuals aged 50 years and older, which in the absence of vaccination, is projected to increase to 19.8 million by 2030.

**Conclusion:**

The model allows for trends in incidence data to be explored based on influential covariates. Incidence rates were shown to vary by age, gender, continent, and over time.

**Funding:**

GlaxoSmithKline Biologicals SA

**Disclosures:**

**Barbara P. Yawn, MD, Msc, ORCID: 0000-0001-7278-5810**, **The GSK group of companies** (Advisor or Review Panel member, Research Grant or Support) **Andrea Callegaro, PhD**, **GSK group of companies** (Employee, Shareholder) **Kyle Fahrbach, PhD**, **Evidera** (Employee)**The GSK group of companies** (Other Financial or Material Support, - KF is employed by Evidera that received financial support by the GSK group of companies during the conduct of the study) **Binod Neupane, PhD**, **Evidera** (Employee)**The GSK group of companies** (Other Financial or Material Support, - BN is employed by Evidera that received financial support by the GSK group of companies during the conduct of the study) **Hilde Vroling, MSc**, **The GSK group of companies** (Research Grant or Support) **Desiree Van Oorschot, Msc**, **The GSK group of companies** (Employee, Shareholder) **Desmond Curran, PhD**, **The GSK group of companies** (Employee, Shareholder)

